# An empirical model of *Drosophila *Photoreceptor-LMC network

**DOI:** 10.1186/1471-2202-16-S1-P47

**Published:** 2015-12-18

**Authors:** Carlos Luna, Daniel Coca

**Affiliations:** 1 Department of Automatic Control and Systems Engineering, University of Sheffield, Sheffield, S101EQ, UK

## 

To operate over the full environmental range of light intensities, fly photoreceptors implement various adaptation mechanisms to continuously adjust their sensitivity. In addition, photoreceptor responses in flies are modulated by feedback from two classes of interneuron, large monopolar cells (LMC) and amacrine cells (AC), and axonal gap-junctions, which pool the responses from six photoreceptors. Previous studies have shown that adaptation in the photoreceptor-interneuron synapses helps extend further the operating range of photoreceptors.

In histamine deficient mutants *hdc*^JK910 ^the lamina interneurons fail to receive and transmit visual information and their feedback synapses can no longer modulate the photoreceptor output. By comparing the 'open-loop' system of mutant flies with the 'closed-loop' system of wild-type flies it is possible to characterize how the network helps enhance the photoreceptor response. We have previously developed a functional nonlinear model of Drosophila's R1-R6 which incorporates separate gains for mean intensity and contrast. By exploiting this control architecture we were able to model the responses of wild type as well as blind mutants using a single model structure and to characterize the contribution from interneurons to boosting the operating ranges of fly photoreceptors.

**Figure 1:**
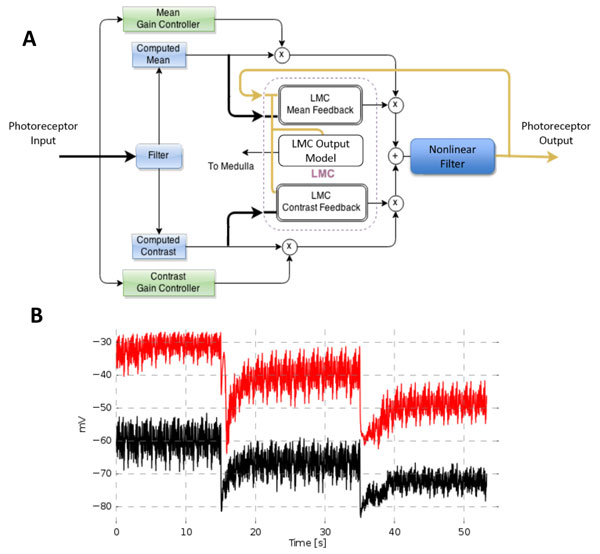
**A. Block diagram of a single photoreceptor-LMC circuit model**. **B**. Experimental recording (black) vs model predicted output (red)

Here we propose a new control structure (see Figure 1 panel A), which separates the gain control implemented by individual photoreceptors from the photoreceptor-LMC feedback-control loop. By exploiting this control architecture we were able to develop an empirical model of *Drosophila *retinal network circuit, which couples six photoreceptor models (R1-R6) with a model of the LMCs. The model not only predicts the individual photoreceptor responses of wild type but also those of *hdc*^JK910 ^mutants by simply disconnecting the LMC feedback loop (see Figure1 Panel B). We used the model to demonstrate the potential role of the retinal circuit in increasing the Signal-to-Noise Ratio of the incoming light stimuli. The elementary circuit model, which was validated extensively using experimental data, can be used to implement a complete spatio-temporal model of the fly's retina.

